# Comparison of Life History Characteristics of the Genetically Modified OX513A Line and a Wild Type Strain of *Aedes aegypti*

**DOI:** 10.1371/journal.pone.0020699

**Published:** 2011-06-17

**Authors:** Irka Bargielowski, Derric Nimmo, Luke Alphey, Jacob C. Koella

**Affiliations:** 1 Division of Biology, Imperial College London, London, United Kingdom; 2 Oxitec Limited, Oxford, United Kingdom; 3 Department of Zoology, University of Oxford, Oxford, United Kingdom; AgroParisTech, France

## Abstract

The idea of implementing genetics-based insect control strategies modelled on the traditional SIT (Sterile Insect Technique), such as RIDL (Release of Insects carrying a Dominant Lethal), is becoming increasingly popular. In this paper, we compare a genetically modified line of *Aedes aegypti* carrying a tetracycline repressible, lethal positive feedback system (OX513A) with a genetically similar, unmodified counterpart and their respective responses to increasing larval rearing density using a constant amount of food per larva. The parameters that we examined were larval mortality, developmental rate (i.e., time to pupation), adult size and longevity.

Analysis revealed some statistically significant differences between the life history traits we examined. The genetically modified OX513A line overall showed 5% lower larval survival as well as reduced adult longevity (20 vs 24 days mean lifespan) compared to the unmodified line. Furthermore, the OX513A line pupated about one day sooner, which could be advantageous in mass-rearing, but produced somewhat smaller adults than the unmodified line; this effect was more pronounced in females than in males.

Increasing the larval rearing density delayed pupation, decreased adult longevity and reduced adult size in both lines. While the delay in pupation and the decrease in longevity were similar between the two lines, the decrease in adult size was more pronounced for OX513A males.

Our study shows that in a controlled laboratory situation the transgenic sterile OX513A line may have somewhat reduced performance compared to its unmodified counterpart and that high rearing densities may further reduce performance. Laboratory-based cage trials as well as field trials are necessary to assess how the differences in life history traits documented here impact the males' success upon release. Furthermore, this paper highlights the potential value of optimisation of mass-rearing systems as optimised rearing methods may be able to alleviate performance issues associated with specific lines or with lab-adapted lines in general.

## Introduction

The development of techniques to transform mosquito species that are vectors of disease, e.g. *Aedes aegypti* [1], [2] and *Anopheles gambiae* [3], has paved the way for new approaches to disease control. One possibility is a genetics-based control strategy modelled on the traditional sterile insect technique (SIT), which uses repressible lethal genes that kill the insect, but which can be repressed to allow rearing of the strain under artificial conditions, i.e. in the laboratory [4].

The RIDL system (release of insects carrying a dominant lethal) is such a strategy [5], [6], [7], [8]. Strains embodying the concept have been engineered for *Ae. aegypti*, using tetracycline-dependent repression of a dominant lethal gene [9], [10]. Tetracycline can be introduced as a dietary supplement for mosquitoes reared in the laboratory, but is not readily available in the wild; hence the lethal system is repressed in the laboratory and activated upon release. Upon their release, transformed males, which are homozygous for this lethal construct, would pass one copy of the dominant lethal to their offspring by normal Mendelian inheritance. These would subsequently die as larvae or pupae in the wild due to the absence of tetracycline. This late-acting lethality, in a species limited by density dependent effects, can be significantly more effective than conventional SIT [9], [11]. Over time, releases of sterile males are expected to reduce the targeted mosquito population.

Releases of mosquitoes, even sterile ones, would preferably be restricted to males, as only female mosquitoes bite – the repeated release of large numbers of females might increase biting nuisance and/or the transmission of disease. Mating opportunities will only present themselves to males that are fit enough and live long enough to successfully compete for habitat, energetic resources and, of course, females. Thus, the performance of male mosquitoes is of paramount importance to sterile-male-release strategies such as RIDL.

The fitness of RIDL insects may be affected by the process of transposon-mediated transformation itself and the subsequent genetic pressures of inbreeding to create a homozygous line [12]; there may also be costs associated with the mass-rearing required to release the large numbers of males that are necessary to make a RIDL (or SIT) programme effective and sustainable. Previous studies have shown that increasing larval density in various mosquito species increases larval mortality, delays pupation and results in smaller, shorter-lived, less fecund adults [13], [14], [15], [16], [17], [18], [19], [20].

In this paper we compare certain life-history traits (larval mortality, developmental rate (i.e. time to pupation), adult size and longevity) of a specific RIDL line, OX513A, carrying a tetracycline repressible, lethal positive feedback system [9], that is considered ready for field release, to those of a closely related, unmodified counterpart. As such, this paper deals with a specific comparison between individual lines, rather than aiming to draw general conclusions about the effects of genetic manipulation. Furthermore, we investigate the effect of increasing rearing density (simulating mass-rearing environments) on the two mosquito lines.

## Results

### Larval survival to pupation

On average, 95% (between 66% and 100%) of the larvae in each pot survived to pupation. Density had no effect on survival (F_2,54_ = 1.32, p = 0.28) but unmodified mosquitoes survived on average about 5% better than the transformed OX513A line (F_1,54_ = 8.01, p = 0.007) (Table 1).

**Table 1:** Results for larval survival, age at pupation, wing length and longevity.

	WT			OX513A		
	1 larvae/ml	4 larvae/ml	8 larvae/ml	1 larvae/ml	4 larvae/ml	8 larvae/ml
**Average larval survival**	94.30%	99.05%	98.74%	92.30%	94.40%	89.71%
	**Males**					
**Average age at pupation**	10.67 (±0.07)	11.19 (±0.04)	10.98 (±0.02)	9.39 (±0.05)	10.45 (±0.03)	10.51 (±0.03)
**Average wing length (mm)**	2.01 (±0.01)	2.03 (±0.01)	1.99 (±0.02)	2.04 (±0.01)	1.94 (±0.01)	1.90 (±0.01)
**Average longevity**	31.60 (±1.43)	24.90 (±1.32)	21.27 (±1.26)	29.3 (±1.47)	19.67 (±1.52)	16.63 (±1.32)
	**Females**					
**Average age at pupation**	11.66 (±0.06)	12.47 (±0.04)	12.27 (±0.03)	9.96 (±0.05)	11.10 (±0.04)	11.35 (±0.03)
**Average wing length (mm)**	2.60 (±0.01)	2.62 (±0.02)	2.31 (±0.01)	2.54 (±0.01)	2.53 (±0.02)	2.28 (±0.01)
**Average longevity**	31.10 (±1.16)	24.90 (±1.11)	21.27 (±0.75)	28.03 (±1.22)	20.60 (±1.05)	16.87 (±1.37)

Comparison of average (n ≥30 (± s.e.m.)) larval survival, age at pupation, wing length and longevity between mosquitoes of the WT and OX513A lines at different larval rearing densities.

### Age at pupation

**Figure 1:**
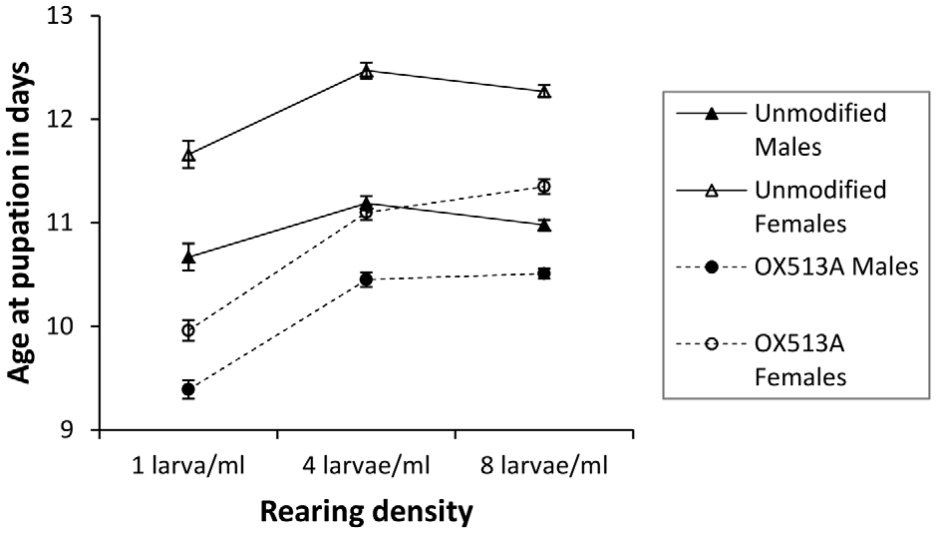
Age at pupation. Comparisons of age at pupation of WT and OX513A mosquitoes reared at different densities in 100 ml pots; error bars showing 95% CI.

Males pupated on average after 10.67 days; females after 11.66 days, a significant difference (F_1,24664_ = 1267, p<0.001); for both, age at pupation ranged from 7 to 18 days. Age at pupation increased by about 1 day from the lowest to the intermediate density, but was similar at the intermediate and the highest density (F_2,54.31_ = 10.8, p<0.001). WT larvae pupated on average about 1 day later than OX513A larvae (F_1,54.34_ = 37.5, p<0.001), and this difference was similar across rearing densities (interaction F_2,54.31_ = 1.58, p = 0.22). The difference between the two lines was less pronounced for males (difference 0.9 days) than for females (difference 1.4 days) (F_1,24648_ = 154, p<0.001) (Table 1, Figure 1).

### Wing length

**Figure 2:**
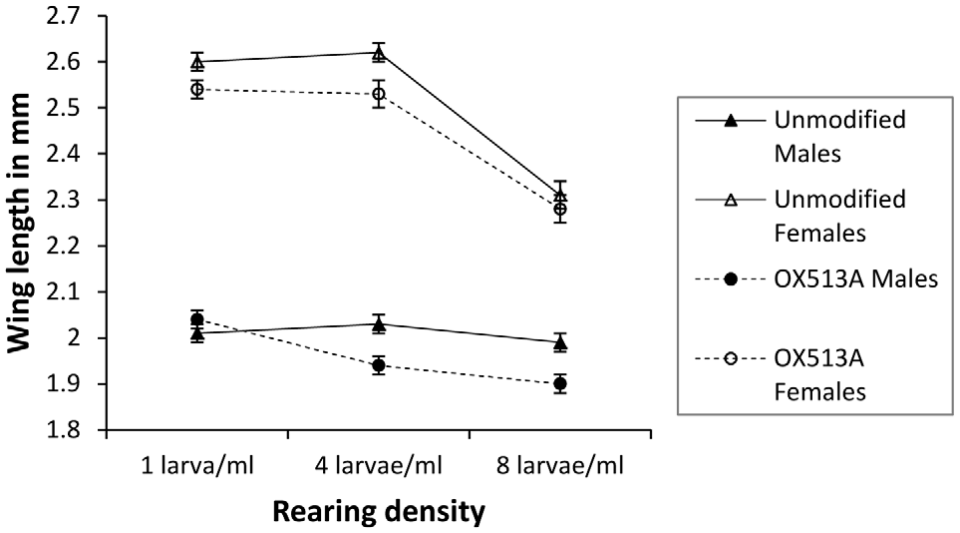
Wing length. Comparisons of average wing length of WT and OX513A mosquitoes reared at different densities in 100 ml pots; error bars showing 95% CI.

Females of both lines were generally larger than the males (F_1, 22.12_ = 3975.57, p<0.001) and showed a greater decrease in wing length with increasing rearing density than the males (F_2,__0.93_ = 83.59, p<0.001). Both male and female unmodified mosquitoes were larger than their OX513A counterparts reared at the same density (F_1, 0.26_ = 46.8, p<0.001). Increased larval rearing density decreased adult wing length for both lines (F_2, 2.32_ = 208.22, p<0.001), but the OX513A line showed a greater response to increasing rearing density (difference 0.204 mm) than the unmodified line (difference 0.155 mm) (F_2, 0.09_ = 8.045, p<0.001), producing increasingly smaller adults. This effect is mainly due to the stronger reaction of OX513A males compared to their unmodified counterparts rather than the females (F_2, 0.08_ = 6.787, p = 0.0013) (Table 1, Figure 2).

### Longevity

**Figure 3:**
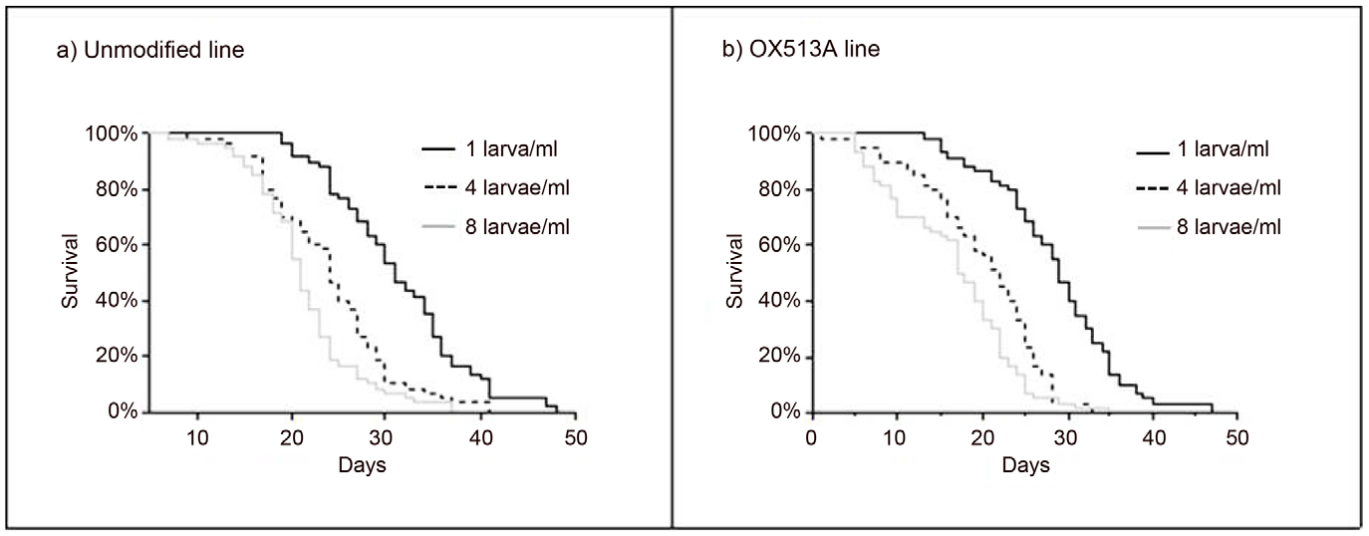
Adult longevity. Survival curves of male WT (a) and OX513A (b) mosquitoes at different rearing densities. All treatments started with 30 individuals. Solid black line representing rearing density of 1 larva/ml; dashed black line representing rearing density of 4 larvae/ml; gray line representing rearing density of 8 larvae/ml.

Adult mosquitoes lived an average of 24 days (1 to 48) irrespective of sex (F_1,348_ = 0.23, p = 0.63). As density increased from 1 to 8 larvae/ml, longevity decreased from 30 days to 19 days (F_2,348_ = 80.1, p<0.001). Unmodified mosquitoes lived about 4 days longer than OX513A transgenics (F_1,348_ = 26.3, p<0.001) (Table 1, Figure 3). None of the interactions were significant (p>0.54).

## Discussion

Our results reveal statistically significant differences between the life history traits of the genetically modified OX513A line and the unmodified line with a similar genetic background. Overall larval survival to pupation was reduced by around 5% in the OX513A line and adult longevity was reduced by about four days. Mosquitoes of the OX513A line pupated on average one day earlier than their unmodified counterparts, with this difference being more pronounced for females (1.4 days) than for males (0.9 day). Perhaps as a consequence, adults of the OX513A line were generally smaller than the unmodified mosquitoes, this difference was again more pronounced for females.

Increasing larval rearing density delayed pupation by approximately one day from the lowest to the intermediate density, but was similar at the intermediate and the highest density in both lines. Moreover, the decrease in adult longevity followed a similar pattern in the two lines with averages decreasing from 30 to 19 days from low rearing density to high rearing density. The average reduction in longevity in response to increased rearing density in either line is larger than the difference between the two lines. Therefore identifying changes in rearing conditions that reduce this negative effect on lifespan are desirable and have the potential to significantly improve male quality for either line. In contrast, the decrease in adult size was significantly different between the two lines with increasing larval rearing density with the OX513A line showing a greater reduction in wing length, especially in males, than the unmodified line.

The reduced time to pupation of OX513A would be advantageous in mass-rearing. However, OX513A adults have a smaller mean size which may be associated with shorter time to pupation. Previous studies show adult body size may play a role in reproductive success. The effect of female body size on reproductive success, for example, is well documented, with there being a direct relationship between body size and oocyte number [21]. Furthermore, Okanda et al. [22], showed that male *Anopheles gambiae* preferentially selected larger females for mating. For males, too, size may play a role. Dickinson and Klowden [23], for example, measured the entire protein content of small vs. large adult male *Aedes aegypti* before and after mating to assess protein transfer. Small males transferred significantly less protein than large males. Ponlawat and Harrington [24] also found an increased sperm capacity in larger males. Yet, how these findings translate into actual fertilization success has not yet been assessed. Research on the effect of male size on mating success in *Anopheles gambiae* – which has different mating habits – [25], [26] concluded that size indeed plays an important role in male competitiveness in this species, with mid to larger sized males being more successful than smaller ones. Although not as clear as the argument for survival and longevity, the smaller size of OX513A mosquitoes may additionally contribute towards a fitness cost compared to unmodified males and should be further investigated. With this in mind, an assessment of the average size of males in any target population is therefore advisable before designing a release programme.

The differences observed in the two lines may be attributable to several possible, non-exclusive factors. The transgenic construct itself may be deleterious by either of two mechanisms. First, the transgene products may have negative effects, i.e. the build-up of alien gene products resulting from the integrated foreign genes may be deleterious to the host cells in which they are expressed. Second, transposition may be associated with insertional mutagenesis, for example a transgene may insert itself in a transcriptionally active region of the genome where it may disrupt native gene function [27], [28], [29].

In the case of OX513A in particular the possibility of non-zero expression of the lethal system (incomplete repression) could play a role. Even low basal ‘leakiness’ of the system could potentially weaken the mosquito.

Furthermore, in these experiments (and after release) adult males of this strain no longer have access to dietary tetracycline. This is likely to derepress the lethal system [30]. Though this clearly does not rapidly kill the males, it is possible that it contributes to their somewhat reduced lifespan relative to wild-type.

These effects may be minimised by suitable design of the construct, but may not be completely eliminated.

Additionally it is likely that fitness is reduced by the inevitable genetic bottleneck associated with starting a new transgenic line from a single transformed individual and the further genetic pressures of inbreeding to make this line homozygous for the lethal gene construct. Most genomes contain numerous recessive mutations that are capable of reducing the fitness of the carrier in a homozygous state [31], [32]. The insertion of a transgene in the vicinity of such a negative recessive mutation and subsequent breeding efforts to make the line homozygous for the inserted construct will at the same time make it homozygous for the recessive allele; this is known as the hitchhiking effect [12] and can lead to the fixation of such alleles causing severe inbreeding depression. This may indeed be one of the most influential factors on the fitness of transgenics, as studies by Amenya et al. [33] and Moreira et al. [34] show that a foreign gene in itself need not negatively impact the fitness of the carrier.

Finally, the two lines should in theory have close to the same genetic background (that of the unmodified line); due to the manner in which the genetically modified mosquitoes were out-crossed with this line to create OX513A. At least 97%–99% of their genome should correspond; nevertheless, it is conceivable that the small amount of the Rockefeller and/or the Mexican genetic background that may remain, especially in the region of the insertion site, is contributing to the differences seen between the lines.

Besides the fitness effects associated with the creation and breeding of the line discussed above that may contribute to the differences between the two lines in this study, environmental factors may also play a role in the fitness of mass-reared mosquitoes when in competition with field-bred mosquitoes in the wild. Environmental stress, such as crowding, during the larval stages of development can, for example, impact the adult mosquito's fitness by reducing its teneral reserves.

Mass rearing environments will contrast with the mosquitoes natural breeding sites in two main respects. Firstly, the larval density in rearing trays will likely be higher than that encountered in natural habitats. Early attempts at mass production of *Ae. aegypti* reared larvae at about 1–2.5 larvae/ml [35], [36], [37], while more recent trials reared larvae at closer to 3–4 larvae/ml (Oxitec Ltd). Being able to rear high numbers of larvae in limited space will be important for the success of control programmes using sterile males. Secondly, in addition to being available in high quantities and in constant supply, the food offered may be of a different composition/quality than that found in natural breeding pools. Therefore, in this study we focus specifically on the effect of larval crowding (i.e. rearing density) on life history traits of genetically modified and wild type mosquitoes, and not on food limitation, as space, not food supply, is considered the most important limiting factor in mass-rearing. Providing each larva with an equal, and sufficient, amount of food daily should eliminate competition for food as a main factor. Nevertheless, as the food was increased in accordance with larval growth, any individuals growing at a slightly faster rate may have eaten proportionately more of the food, to some extent creating competition for food over time.

Moreover, the high larval density will result in large quantities of waste materials such as dead and decomposing larvae, discarded exoskeletons, excretory products and surplus food entering the system. Consequently, as the larvae age the water quality will degrade unless it is changed regularly. Growing in polluted water can have a negative impact on larval development [38]. The possible contaminants in high-density rearing water include allelopathic substances, such as a growth retardant, synthesised in reaction to competitive stress, excretory products such as nitrogenous waste as well as food waste or larval debris, or bacterial growth. These may have a direct effect on larval growth, or indirect effects, for example *via* effects on microbial composition of the habitat.

Early work on larval crowding [39], [40], [41] suggested the existence of a growth retardant factor excreted by mosquito larvae under intraspecific competition, especially when food became limiting. However, other studies found no such growth retardant factor [17]. Therefore, the existence of such products is still questionable and the contribution they may make to intraspecific competition undefined, indeed no such product has been identified for any other animal species [38].

Recent results for various mosquito species, *Aedes albopictus*, *Tripteroides bambusa* [42] and *Aedes aegypti* [38], demonstrate a negative effect of rearing mosquitoes in water that has already been occupied by a previous batch of larvae. The latter experiment in particular is of interest as mosquitoes were reared individually, thus eliminating the element of competition and therefore any need to produce growth retardants. Furthermore, studies on *Aedes triseriatus* [43] showed the accumulation of ammonia in tree-holes occupied by larvae, while Carpenter [44] showed that the addition of ammonia to microcosms containing *Ae. triseriatus* had negative effect on survival and development as well as adult mass.

Finally, it is possible that bacterial or fungal growth could affect life history parameters in mass rearing trays. Depending on the microbial community present in the rearing water this can either benefit larval development as some bacteria acts as an additional food source [45], while some fungal species can negatively impact larval development as shown by Mokany and Shine [46]. Furthermore, the micro-biota could indirectly affect the larvae by contaminating their food supply, rendering it less nutritious or even inedible.

As all the factors described above will increase with increasing number of larvae per millilitre the negative impact water pollution may have will increase accordingly, in line with our results, potentially leading to later pupation and smaller, shorter lived adults.

### Conclusions

Assessing life-history traits is only one part of fitness. As a complementary study (in prep) we aim to compare the fitness of the males in competition for females and, in particular, their mating success.

Nevertheless, despite possible complications, our study shows that in a controlled laboratory situation the OX513A line may have somewhat reduced performance compared to its unmodified counterpart and that high rearing densities necessarily associated with mass-rearing may further reduce performance. Such potential reduction in performance must however be confirmed in the field as laboratory-based and field-based trials do not always show similar effects [47], [48], [49]. Furthermore, the unmodified line used in these experiments has been reared under laboratory conditions for many generations. It may therefore be significantly lab-adapted and/or inbred and may differ substantially from any target field population [50]. Field populations may also differ due to environmental factors. Consequently, the modified males may face different and perhaps ‘tougher’ competition upon release than these laboratory trials can simulate. Differences detected between the lines here should therefore be treated as conservative estimates.

Simulation models may be useful to explore the impact of line performance on the effectiveness of any future control programme using such lines. It is likely the modest performance reduction indicated here for OX513A relative to an unmodified strain could be compensated by releasing more males. However, our data indicate there may be some scope for improvement in the construction of future strains. Furthermore, this paper highlights the potential value of optimisation of mass-rearing systems as optimised rearing methods may be able to alleviate performance issues associated with specific lines or with lab-adapted lines in general to a certain extent. Unfortunately, advances in mosquito mass-rearing have in recent years lagged far behind advances in mosquito genetics.

## Methods

### Mosquito strains

#### Unmodified line

The unmodified line analysed in this paper originates from field caught *Aedes aegypti* from Jinjang, Selangor, Malaysia. It was originally colonised in 1975 and has since been held in the laboratory. It can therefore be considered a highly lab-adapted strain and is not necessarily representative of field bred males; however it was chosen because of its genetic similarity to the modified OX513A line.

#### RIDL line (OX513A)

OX513A is a homozygous RIDL line of *Ae. aegypti*, transformed with a tetracycline repressible, lethal positive feedback system [9]. A tetracycline-repressible transcriptional transactivator (tTAV) [30], [51] under the control of its own binding site (tetO) creates a positive feedback loop. The addition of tetracycline leads tTAV to bind tetracycline, in which form tTAV can no longer bind to tetO and the cycle is interrupted [9].

Mosquitoes of this line are identifiable by red fluorescence due to the expression of DsRed2 under the control of an Act5C promoter [9].

The OX513A line was originally created in the Rockefeller strain and subsequently out-crossed into a Mexican line of *Aedes aegypti*. It has since been out-crossed to the unmodified line described above for five generations in such a fashion that at least 97%–99% of their genome should correspond. Selection of homozygous individuals was initially based on intensity of fluorescence and then confirmed by PCR after mating/laying. To minimize the effects of inbreeding, 44 independent homozygous females were combined with homozygous males and pooled to create the OX513A homozygous strain used here.

### Experimental design and larval rearing

All experiments were conducted in a temperature-controlled insectary at 27 (+/−2) °C and a relative humidity of 65 (+/−10)% with a 12 h:12 h light/dark cycle.

Eggs of the unmodified *Aedes aegypti* line and the OX513A line were submerged in water supplemented with tetracycline to a final concentration of 30µg/ml and placed under low pressure for one hour to ensure synchronous hatching. The following day, larvae were counted out into 100 ml pots of tap water (surface area of the water: 79 cm, water depth 1.5 cm) at densities of 100, 400 and 800 larvae per pot, thus giving rearing conditions of 1, 4 and 8 larvae/ml.

The larvae were fed the following feeding regime of finely ground TetraMin fish food per larva: day 1–0.03 mg, day 2– no food, day 3–0.04 mg, day 4–0.08 mg, day 5–0.16 mg, day 6–0.16 mg, day 7 onwards –0.32 mg. Rearing was carried out in four consecutive blocks staggered by three days. This blocked design was repeated three times giving a total of 30 pots per treatment.

Pupae were removed from pots by pipette on the day of pupation and their numbers and sex recorded.

One male and one female pupae of each rearing pot (so 30 mosquitoes per treatment) were moved into individual pots to eclose. Emerged adult mosquitoes were supplied with a piece of cotton wool saturated with a 10% sucrose solution, which was refreshed every other day to prevent desiccation. Survival was recorded daily.

The other mosquitoes were frozen and their wing length was measured. Wings were removed in a 70% ethanol solution under a dissection microscope and mounted on microscope slides. Digital images of the wings alongside a graticule for purposes of scale were taken using a Canon PowerShot S5IS camera and a 99 mm adapter (S/N:3754, Martin Microscope Company). Wings were measured with ImageJ (http://rsbweb.nih.gov/ij/) from the auxiliary incision to the apical margin excluding the fringe.

### Statistical analysis

Statistical analyses were performed with JMP version 7.0 (http://www.jmpdiscovery.com). Age at pupation and wing length were analyzed with mixed effect anovas including density, line, sex and up to 2-way interactions (the 3-way interaction was not significant) as fixed factors and pot as a random factor nested within density and line. Larval survival was estimated as the proportion of individuals surviving to pupation in each pot, was Box-Cox transformed, and was analysed as an anova including line, density and their interaction. Adult longevity was analysed as an anova including density, line, sex and their interactions. We used an anova instead of a survival analysis, as no mosquitoes were censored and the distribution of longevity was close to normal. However, a survival analysis (Kaplan-Meier) gave similar results (not shown). As density must be a nominal factor in the nested analysis, to ensure consistency, it was considered nominal in all analyses.
